# Efficacy and safety of different doses of epidural morphine coadministered with low-concentration ropivacaine after cesarean section: A retrospective cohort study

**DOI:** 10.3389/fphar.2023.1126174

**Published:** 2023-04-06

**Authors:** Liang Sun, Shuo Guan, Dou Dou, Yi Feng, Hong Zhang, Haiyan An

**Affiliations:** Department of Anesthesiology, Peking University People’s Hospital, Beijing, China

**Keywords:** cesarean section, epidural analgesia, morphine, maternal outcomes, urinary retention

## Abstract

**Objective:** The optimal dose of epidural morphine after cesarean section (CS) still remains unknown when combined with low-concentration ropivacaine based on a continuous basal infusion (CBI) mode. The aim of this study was to assess the impact of different dose of epidural morphine plus ropivacaine on maternal outcomes.

**Materials and methods:** Data of parturients who received epidural analgesia for CS at a teaching hospital from March 2021 to June 2022 were retrospectively collected. Parturients were divided into two groups (RM3 group and RM6 group) according to different medication regimens of morphine. The implementation of epidural analgesia was performed with 3 mg morphine in RM3 group and 6 mg morphine in RM6 group in combination with 0.1% ropivacaine *via* a CBI pump. The primary outcomes included pain intensity at rest and movement and the incidence of urinary retention and pruritus within postoperative 48 h. The secondary outcomes included the incidence and severity of postoperative nausea and vomiting (PONV) and pruritus, the rate of rescue analgesia and grading of motor Block.

**Results:** Totally, 531 parturients were eligible for the final analysis, with 428 and 103 parturients in the RM3 group and RM6 group, respectively. There were no statistically significant differences in the visual analogue scores (VAS) at rest and movement within postoperative 48 h between the two groups (all *p* > 0.05). Compared with the RM6 group, the incidence of urinary retention was lower in the RM3 group within 48 h after CS (4.0% vs. 8.7%, *p* = 0.044). No significant difference was found in the incidence and severity of PONV and pruritus, the rate of rescue analgesia and grading of motor block between RM3 and RM6 groups.

**Conclusion:** Epidural 3 mg morphine plus 0.1% ropivacaine in a CBI mode can provide equal efficacy and have lower incidence of urinary retention compared with 6 mg morphine after CS.

## Introduction

A cesarean section (CS) remains the most commonly performed procedure in the obstetric settings worldwide. However, pain after CS is a common problem, and moderate to severe pain has been reported in a large proportion of parturients (Ryu et al., 2022). Establishing and maintaining adequate pain control after CS with minimal adverse reactions facilitates a rapid recovery to ambulate and baby care for parturients.

Combined spinal-epidural anesthesia (CSEA) contributes as an advantageous anesthetic technique for CS considering that epidural catheter can concomitantly provide postoperative analgesia for parturients. However, epidural analgesia with local anesthetics alone such as ropivacaine frequently leads to lower extremity numbness and weakness in a time-dependent and dose-dependent manner, especially using a high concentration, thereby delaying early postoperative recovery (Suzuki et al., 2015). Alternatively, epidural opioid (e.g., morphine) alone after CS can provide excellent postoperative pain relief and has a low rate of lower extremity motor block (Donchin et al., 1981; Chumpathong et al., 2002; Fonseca et al., 2020). Unfortunately, epidural opioid (e.g., morphine) analgesia could be associated with complications, including postoperative nausea and vomiting (PONV), pruritus, respiratory depression, etc., which hinder early recovery after CS (Guasch et al., 2020). Multiple studies have demonstrated that analgesia is more effective and some annoying side effects are minimized when opioids are administered in combination with local anesthetics (Yang et al., 2019; Miao et al., 2021; Otao et al., 2021). Notably, this multimodal approach has been shown to provide superior analgesia to that of intramuscular or intravenous administration of opioids without significant side effects (Stocki et al., 2014; Yang et al., 2019).

Due to its high degree of hydrophilic properties, epidural morphine could provide more effective analgesia with slow onset and longer duration, and thus has been frequently used in different clinical scenarios (Mercanoğlu et al., 2013; Meng et al., 2019; Yang et al., 2019; Abdelemam et al., 2022). However, the aforementioned opioid-related adverse events remain evitable. Therefore, some reports have indicated that these side effects may be related to the doses of opioids that are administered and advocated using a modified regimen, such as different combinations of local anesthetics with opioids, to decrease dosage of both opioid and local anesthetics, and thus reduced the incidence of disturbing side effects (Chen et al., 2011; Yang et al., 2019; Oshima and Aoyama, 2022). However, current regimens that contain even small doses of epidural opioids still cause those complications, which are clinically bothersome to many patients (Yang et al., 2019; Oshima and Aoyama, 2022). Therefore, the optimal dose of epidural opioid including morphine which provides potent analgesia but with minimal adverse reactions remains undetermined.

Unlike other centers, as a regular protocol, epidural analgesia was performed with 0.1% ropivacaine in combination with morphine *via* an automatic continuous basal infusion (CBI) pump after CS for many years in our center. The dose of morphine continued to decrease over time, and the efficacy and safety of different doses of epidural morphine coadministered with low-concentration ropivacaine has long been observed and recorded. However, the optimal dose of epidural morphine in such mode still remained unresolved.

Therefore, based on previous clinical experience and data, it is prudent to perform a retrospective cohort study to compare the efficacy and safety of epidural 0.1% ropivacaine coadministered with 3 mg versus 6 mg after CS, which was the highest and lowest morphine dose respectively in our center by far, and optimize future analgesia regimen.

## Materials and methods

This retrospective cohort study was approved by the institutional review board of Peking University People’s Hospital (approval number: 2022PHB016-001). All the study protocol was carried out in accordance with the ethical standards of the Declaration of Helsinki and its subsequent amendments.

**FIGURE 1:**
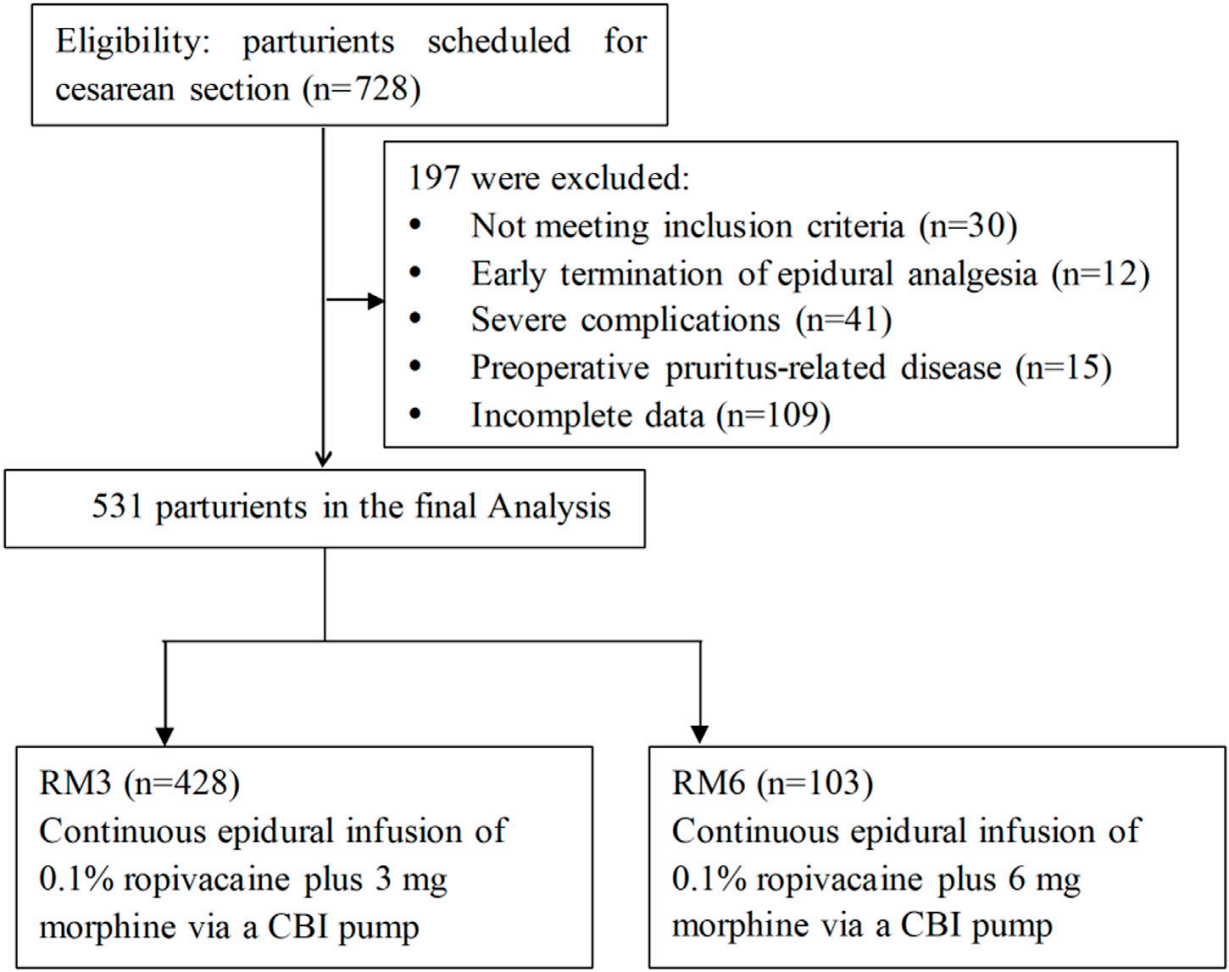
The flow diagram of the study protocol. CBI, continuous basal infusion.

Medical records of patureints aged 21–45 years old who underwent scheduled CS and received postoperative epidural analgesia were retrospectively reviewed and collected from July 2021 to June 2022. Exclusion criteria included 1) serious cardiovascular disease, pulmonary disease and liver and kidney dysfunction before CS, 2) early termination of epidural analgesia due to severe intolerance, accidental withdrawal of the catheter, or equipment failure during epidural analgesia, 3) dermatitis, eczema and skin infection which caused pruritus before CS, 4) preoperative urinary retention or urinary tract obstruction, 5) lower limb motor and sensory disorders before CS and 6) incomplete data. The flow diagram of the study was presented in Figure 1.

Parturients were classified into two groups: parturients who received epidural 0.1% ropivacaine plus 3 mg morphine (RM3 group) and parturients who received epidural 0.1% ropivacaine plus 6 mg morphine (RM3 group). Both regimens were performed *via* an automatic CBI pump without patient-controlled function, with the infusion speed of 2 mL/h. The analgesic formula was consisted of 0.1% ropivacaine with 0.03 mg/mL or 0.06 mg/mL in the RM3 or the RM6 group, respectively.

Demographic characteristics were collected, including the maternal age, gestational weeks, parity, height, weight, BMI, history of CS, pregnancies. Clinical data were also collected within 48 h after CS and included pain intensity, rescue analgesic use, and analgesia-related adverse events [lower extremity numbness and weakness, postoperative nausea and vomiting (PONV), urinary retention and pruritus]. All the data were collected from the electronic medical record system of our hospital.

The primary outcomes included pain intensity at rest and movement and the incidence of urinary retention and pruritus within postoperative 48 h. Pain intensity was evaluated by using visual analog scale (VAS), with “0” representing no pain and “10” the worst pain (Maged et al., 2018). Urinary retention was defined as the inability to void 6 h after urethral catheter removal that will require catheterization to obtain relief (Igbodike et al., 2021). The pruritus severity of scalp was evaluated according to the VAS method (Li et al., 2022). The secondary outcomes included the incidence and severity of PONV and grading of motor block. The modified Bromage scale was used to grade the motor block associated with epidural analgesia. The grading was done as 0: no motor block, 1: inability to raise extended legs but able to move knees and feet, 2: inability to raise extended legs and move knees but able to move feet, and 3: motor limb was completely blocked (Cheng et al., 2019). Nausea and vomiting were rated in terms of incidence and severity over the postoperative 48 h period using the following scoring system: 0, side effect not experienced; 1, side effects experienced, no treatment needed; 2, side effect experienced treatment effective; 3, side effect experienced, and treatment ineffective (Yayla et al., 2022).

The data are presented as the mean ± standard deviations (SDs) for normally distributed continuous variables or median (minimum, maximum) if distributions were skewed, while categorical variables are expressed as frequencies or percentages. Normal distribution of the data was tested using the Kolmogorov Smirnov test. Independent sample *t*-test or Mann-Whitney U test was performed between the groups. The comparison of categorical data used the χ2 test. All data were analyzed using SPSS 19.0 statistical software package (SPSS, Inc., Chicago, IL, United States), a *p* < 0.05 was considered statistically significant.

## Results

### Demographic and clinical characteristics of included paturients

Initially, data of 728 paturients were retrospectively collected and reviewed. According to the inclusion and exclusion criteria, a total of 531 paturients during the study period were included in the final analysis, with 103 parturients in the RM6 group and 428 parturients in the RM3 group, respectively.

The demographic and clinical characteristics including the maternal age, height, weight, BMI, gestational weeks, rate of nulliparity, proportion of singleton, and history of CS did not demonstrate significant differences between the RM3 group and the RM6 group (all *p* > 0.05) (Table 1).

**TABLE 1:** Demographic and clinical characteristics of parturients.

Parameters	RM3 group	RM6 group	t/χ^2^/Z	*p* value
	(n = 428)	(n = 103)		
Age (years)	33.8 ± 4.0	34.4 ± 4.0	−1.281	0.201
Height (cm)	161.8 ± 5.6	162.8 ± 5.0	−1.622	0.105
Weight (kg)	73.0 ± 11.2	75.1 ± 10.9	−1.744	0.082
BMI (kg/m^2^)	27.9 ± 4.0	28.4 ± 3.8	−1.071	0.285
Gestational weeks (week)	38.3 ± 2.1	38.7 ± 2.2	−1.725	0.085
Nulliparity n (%)	284 (66.4%)	58 (56.3%)	3.654	0.056
Singleton n (%)	405 (94.6%)	100 (97.1%)	1.080	0.299
History of CS n (%)	113 (26.4%)	25 (24.3%)	0.196	0.658

Data are expressed as mean± standard deviations (SDs) or frequencies (percentages). CS, cesarean section.

### Efficacy of analgesia

As shown in Table 2, no significant difference was detected in the VAS score both at rest [1 (0–5) vs. 1 (0–5), *p* = 0.394] and movement [3 (0–7) vs. 3 (1–7), *p* = 0.938] between RM3 and RM6 groups on POD 1, as well as on POD 2 [0 (0–3) vs. 0 (0–3) at rest, *p* = 0.935; 2 (0–5) vs. 2 (0–5) at movement, *p* = 0.985]. Meanwhile, the frequency of rescue analgesic use within 48 h was not significantly different between the groups [66 (15.4%) vs. 12 (11.7%) on POD1, *p* = 0.332; 11 (2.6%) vs. 3 (2.9%) on POD2, *p* = 0.846] (Table 2).

**TABLE 2:** Analgesic efficacy and rescue analgesia in RM3 and RM6 groups.

Parameters	RM3 group	RM6 group	t/χ^2^/Z	*p* value
	(n = 428)	(n = 103)		
VAS at rest on POD1	1 (0–5)	1 (0–5)	−0.852	0.394
VAS at movement on POD1	3 (0–7)	3 (1–7)	−0.077	0.938
Rescue analgesia on POD1 n (%)	66 (15.4%)	12 (11.7%)	0.942	0.332
VAS at rest on POD2	0 (0–3)	0 (0–3)	−0.082	0.935
VAS at movement on POD2	2 (0–5)	2 (0–5)	−0.018	0.985
Rescue analgesia on POD2 n (%)	11 (2.6%)	3 (2.9%)	0.038	0.846

Data are expressed as mean± standard deviations (SDs) or median (minimum, maximum). VAS, visual analogue scare; POD, postoperative day.

### Incidence and severity of pruritus

There was no significant difference in the incidence of pruritus both on POD1 [54 (12.6%) vs. 14 (13.6%), *p* = 0.071] and POD2 [22 (5.1%) vs.7 (6.8%), *p* = 0.441] between RM3 and RM6 groups. Likewise, the VAS score of pruritus within 48 h was not significantly different between the groups [0 (0–3) vs. 0 (0–3) on POD1, *p* = 0.377; 0 (0–3) vs. 0 (0–2) on POD2, *p* = 0.623] (Table 3).

**TABLE 3:** Incidence, severity score, and distribution of reported motor weakness and side effects in the RM3 and RM6 groups.

Parameters	RM3 group	RM6 group	t/χ^2^/Z	*p* value
	(n = 428)	(n = 103)		
Pruritus score on POD1	0 (0–3)	0 (0–3)	0.377	0.706
Pruritus on POD1 n (%)	54 (12.6%)	14 (13.6%)	0.071	0.790
nausea score on POD1	0 (0–2)	0 (0–3)	1.516	0.130
Nausea on POD1 n (%)	15 (3.5%)	7 (6.8%)	2.265	0.132
Vomiting on POD1 n (%)	4 (0.9%)	1 (1.0%)	0.001	0.973
Pruritus score on POD 2	0 (0–3)	0 (0–2)	0.623	0.533
Pruritus on POD2 n (%)	22 (5.1%)	7 (6.8%)	0.441	0.507
nausea score on POD2	0 (0–1)	0 (0–1)	−0.170	0.865
Nausea on POD2 n (%)	5 (1.2%)	1 (1.0%)	0.029	0.865
Vomiting on POD2 n (%)	0 (0%)	0 (0%)	-	-
Urinary retention n (%)	17 (4.0%)	9 (8.7%)	4.049	0.044

Data are expressed as mean± standard deviations (SDs) or median (minimum, maximum). POD, postoperative day.

### Incidence of urinary retention

As shown in Table 3, less parturients in RM3 group experienced urinary retention within postoperative 48 h after CS, compared with RM6 group [4.0% (17/428) vs. 8.7% (9/103), *p* = 0.044].

### Incidence and severity of PONV

No significant difference in the incidence of PONV both on POD1 [nausea: 15 (3.5%) vs. 7 (6.8%), *p* = 0.132; vomiting: 4 (0.9%) vs. 1 (1.0%), *p* = 0.973] and POD2 [nausea: 5 (1.2%) vs.1 (1.0%), *p* = 0.865] was detected between RM3 and RM6 groups. Moreover, no case of vomiting was detected in both groups on POD2. Similarly, the severity of nausea within 48 h was not significantly different between the groups both on POD1 [0 (0–2) vs. 0 (0–3) on POD1, *p* = 0.130] and POD 2 [0 (0–1) vs. 0 (0–1) on POD2, *p* = 0.865] (Table 3).

### Rate of motor block

No motor block was reported both in RM3 and RM6 groups within 48 h after CS.

## Discussion

Low-concentration of ropivacaine coadministered with opioid including morphine has been a common protocol used in epidural analgesia after CS (Zhang et al., 2021; Wang et al., 2022). Adding morphine to epidural local anesthetics can effectively enhance the analgesia effect and concomitantly reduce the incidence of adverse reactions caused by local anesthetics. However, there are concerns of the opioid-related adverse effects on the maternal outcomes (Lu et al., 2018; Miao et al., 2021). In fact, both 3 mg and 6 mg epidural morphine were used in combination with 0.1% ropivacaine *via* CBI pump after CS in our center. Therefore, it is clinically beneficial to determine the optimal dose of epidural opioid when adding to low-concentration of local anesthetics. Herein, our retrospective study demonstrated that compared with 6 mg morphine, 3 mg morphine in combination with 0.1% ropivacaine *via* a CBI pump for epidural analgesia after CS can provide non-inferior analgesia efficacy. Moreover, the incidence of epidural morphine-related urinary retention was significantly decreased though no differences in other morphine-related adverse reactions were detected, suggesting that 3 mg morphine is more advantageous than 6 mg morphine as the supplementation to the epidural ropivacaine and conducive to facilitating the fast recovery of paturients after CS.

The increasing use of CSEA adds the possibility of the epidural administration of a local anesthetic after CS, such as bupivacaine, after spinal anesthesia. Ropivacaine is more recommended for little influence on the hemodynamics, shorter duration of sensory block and motor block and low incidence rate of adverse reactions, which facilitates the recovery and thus epidural ropivacaine has been suggested as superior to bupivacaine for postoperative pain control (Fischer et al., 2000; Aşik et al., 2002; Wang et al., 2019). The literature has shown that the use of 0.1% ropivacaine as epidural analgesia can provide preferable analgesic potency, and adverse effects becomes more frequent with increasing of the concentration of ropivacaine, especially the motor blockade (Miao et al., 2021; Otao et al., 2021; Zhang et al., 2021). In our study, no lower limb motor weakness was reported in any case, which was in line with previous studies. Adding epidural opioids including morphine following CS has been used clinically in recent decades with excellent analgesic effects, but it has bothersome side effects, such as pruritus, PONV, urinary retention, which are difficult to prevent, and gradually leading to the dissatisfaction of the parturients (Sultan et al., 2016; Payne et al., 2021). Moreover, many studies reported that these side effects were dose-dependent and higher dose of epidural opioids were often associated with more frequent potential adverse events (Otao et al., 2021; Oshima and Aoyama, 2022). However, another study demonstrated that adverse effects were comparable in different doses of epidural hydromorphone coadministered with ropivacaine after CS (Yang et al., 2019). Our study revealed that the incidence of urinary retention was lower in RM3 group compared with RM6 group, but other adverse events were similar between the two groups.

Contrary to patient-controlled epidural analgesia (PCEA) performed in other centers, analgesia regimen after CS in our center used epidural morphine and 0.1% ropivacaine *via* a CBI pump. The current study revealed that this mode can provide effective analgesia with acceptable side effects. In fact, the literature indicated that CBI mode was similar to PCEA or automated mandatory bolus (AMB) for maintaining epidural analgesia for labor or after CS in terms of all measured maternal and fetal outcomes, though the later ones may have the benefit of decreasing the risk of breakthrough pain and improving maternal satisfaction (Sng et al., 2018). However, compared with the PCEA mode, epidural opioid and low-concentration of local anesthetics *via* a CBI pump needs less physician workload and nursing personnel. Additionally, severe complications can be avoided due to misprogramming occurred in using the PCEA device. Therefore, current performed epidural analgesia regimen performed in our center is a relatively preferable method for pain management after CS.

Our study has several limitations. First, this was a retrospective single-center cohort study, and thus there may be some selection bias. Second, morphine and low-concentration of ropivacaine regimens for epidural analgesia were commonly used in our single institution, which might not be popularized entirely to other centers. Third, due to the nature of the retrospective study, some unknown or unmeasured confounders, and those excluded parturients who had incomplete data may interfere with the outcomes. Therefore, our results should be extrapolated cautiously.

## Conclusion

In conclusion, the current study demonstrated that 3 mg morphine plus 0.1% ropivacaine for epidural analgesia *via* a CBI pump can provide equal efficacy and have lower incidence of urinary retention compared with 6 mg morphine after CS. Future multicenter randomized controlled trials are warranted to determine the optimal dose of epidural morphine in CBI mode with better safety profile when administered with concurrent low-concentration of ropivacaine.

## Data Availability

The raw data supporting the conclusion of this article will be made available upon reasonable request to the corresponding authors.
